# Genetic Aspects of Somatic Cell Count in Holstein Dairy Cows in Iran

**DOI:** 10.3390/ani11061637

**Published:** 2021-06-01

**Authors:** Hadi Atashi, Miel Hostens

**Affiliations:** 1 Department of Animal Science, Shiraz University, Shiraz 7155713876, Iran; 2 Department of Farm Animal Health, University of Utrecht, Yalelaan 7, 3584 CL Utrecht, The Netherlands; m.m.hostens@uu.nl

**Keywords:** genetic parameter, random regression model, Holstein

## Abstract

**Simple Summary:**

Holstein Friesian is the dominant breed of modern dairy cattle in Iran, therefore the development of a genetic evaluation system for economically important traits for this population is needed. Genetic parameters for SCS and its genetic correlations with production traits were estimated in Iranian Holstein cows. The moderate heritability for SCS and its low negative genetic correlations with yield traits indicate that genetic selection for decreasing SCS would have a relatively medium genetic progress with no necessarily antagonistic effects on lactation performance. The estimates found in this study can be considered as the first step to include SCS in the national genetic evaluations in Iranian Holsteins using a multiple-trait, multiple-lactation random regression model.

**Abstract:**

The aim of this study was to estimate the genetic parameters of somatic cell count (SCC) and its relationship with production traits in the first three parities in Iranian Holstein dairy cows. Data were 1,891,559 test-day records of SCC, milk yield, and milk compositions on 276,217 lactations on 147,278 cows distributed in 134 herds. The number of test-day records in the first, second and third parities were 995,788 (on 147,278 cows), 593,848 (on 85,153 cows), and 301,923 (on 43,786 cows), respectively. Test-day SCCs were transformed to somatic cell scores (SCS). A random regression test-day animal model through four-trait three-lactation was used to estimate variance components for test-day records of SCS and lactation traits were included. Gibbs sampling was used to obtain marginal posterior distributions for the various parameters using a single chain of 200,000 iterates in which the first 50,000 iterates of each chain were regarded as a burn-in period. The mean heritability estimates for SCS (0.15 to 0.18) were lower than those for milk yield (0.36 to 0.38), fat yield (0.30 to 0.31), protein yield (0.31 to 0.32), fat percentage (0.21 to 0.25), and protein percentage (0.21 to 0.22). Low negative genetic correlations ranging from −0.05 to −0.30 were found between SCS and yield traits (milk, fat, and protein yields). The genetic correlation found between SCS and fat percentage was close to zero, however, a low positive genetic correlation ranging from 0.12 to 0.17 was found between SCS and protein percentage. Based on the results, it can be concluded that genetic selection for decreasing SCS would also increase lactation yield. The estimates found in this study can be used to perform breeding value estimations for national genetic evaluations in Iranian Holsteins using a multiple-trait, multiple-lactation random regression model.

## 1. Introduction

There are about seven million cattle in Iran, of which some 700,000 are Holstein distributed in more than 2000 herds. The herd sizes vary from 20 to 3000 dairy cows with a mean 305-day milk production of 7200 kg [1,2]. Although the use of selection indices has been a common practice during the last decades [3,4], in Iran, bull and cow selection is mainly based on the estimated breeding value of milk yield. The only nationally devised genetic index in use includes the production traits and longevity [2]. However, milk price in Iran is based on bulk tank somatic cell count (SCC) thereby affecting revenues. Somatic cell score (SCS), the average of the log of test-day SCC, could be included in the economic breeding index as a proxy for udder health. However, accurate genetic parameters for the prevailing population are required for any trait to be included in a selection index. Different models (e.g., repeatability model, random regression, or fixed regression test-day model) are used for the national genetic evaluation of SCS in Interbull member countries [5]. The first random regression test-day (RR-TDM) model for national genetic evaluation of dairy cattle was adopted at the beginning of the 21st century [6]. Since then, RR-TD models have become the models of choice for genetic evaluation of production traits in dairy cattle [7,8]. The RR-TD model was subsequently adopted for national genetic evaluation of dairy breeds, and most of the countries that participated in the international evaluations for production traits of Holstein bulls used a test-day model for national genetic evaluations, while others used a lactation animal model [5]. Holstein Friesian is the dominant breed of modern dairy cattle in Iran, therefore the development of a genetic evaluation system for economically important traits for this population is needed. The objective of the present study was to estimate genetic parameters for SCS and its genetic correlations with production traits in the first three parities in Iranian Holstein cows using a multiple-trait, multiple-lactation random regression test-day model.

## 2. Materials and Methods

### 2.1. Data

Data used in this study were test-day records from Holstein cows collected between January 2009 to December 2018 by the Animal Breeding Center of Iran (Karaj, Iran, 35°49′ N and 50°59′ E). All evaluated herds were purebred Holsteins, managed under conditions similar to those used in most developed countries, and were under official performance and pedigree recording. The diet, fed as a total mixed ration (TMR), mainly consisted of corn silage, alfalfa hay, barley grain, fat powder, beet pulp, and feed additives. Monthly milk recording was performed by trained technicians of the Iranian Animal Breeding Center, according to the guidelines of the International Committee for Animal Recording [9]. Cows with missing birth dates, calving dates, or lactation numbers were excluded. Only records from the first three parities that had data for SCC, milk yield, and milk compositions on a given test-day remained in the dataset. Records from days in milk (DIM) lower than 5 and greater than 365 were eliminated. Within cows, if lactation number three was present, lactations number one and two were also present, and if lactation number two was present, lactation number one was also present. Age at the first calving (AFC) was calculated as the difference between birth date and calving date at the first parity and restricted to the range of 640 to 1200 d. Edited data consisted of 1,891,559 test-day records of milk yield (MY), fat yield (FY), protein yield (PY), fat percentage (FP), protein percentage (PP), and somatic cell count (SCC) on 276,217 lactations on 147,278 cows in 134 herds. The number of test-day records in the first, second, and third parity were 995,788 (on 147,278 cows), 593,848 (on 85,153 cows), and 301,923 (on 43,786 cows), respectively. Full pedigree records were included from 386,955 females and 4219 males. Test-day SCCs were transformed into somatic cell scores (SCS) based on the following equation: $\mathrm{SCS}=\log_{2}\left(\mathrm{SCC}/100,000\right)+3$.

### 2.2. Model

The following random regression (RR) test-day animal model through four-trait three-lactation was used to estimate variance components for test-day records of MY, FY, PY, FP, PP, and SCS:

(1) $$y_{\mathrm{ijklm}}=\;µ\;+{\;\mathrm{HTDp}}_{i}+\overset{4}{\underset{b=0}{\sum }}{\mathrm{AS}}_{j}{ø}_{b}\left(t\right)+\overset{2}{\underset{b=0}{\sum }}a_{l}{ø}_{b}\left(t\right)+\overset{2}{\underset{b=0}{\sum }}{\mathrm{pe}}_{l}{ø}_{b}\left(t\right)+{\;e}_{\mathrm{ijklm}}$$

where $y_{\mathrm{ijklm}}$ is the test-day record (MY, FY, PY, FP, PP, and SCS) on DIM m of cow l in parity k, belonging to i^th^ class of HTDp, and j^th^ class of AS, ${\mathrm{HTDp}}_{i}$ is the fixed effect of i^th^ class of herd-test-day parity, ${\mathrm{AS}}_{k}$ is the fixed effect of age-season of calving defined as the following: age at calving (five intervals of age at calving were created for each parity) × season of calving (four seasons: winter from Jan–Mar, spring from Apr–Jun, summer from Jul–Sep and autumn from Oct–Dec), $\overset{2}{\underset{b=0}{\sum }}a_{l}{ø}_{b}\left(t\right)$ and $\overset{2}{\underset{b=0}{\sum }}{\mathrm{pe}}_{l}{ø}_{b}\left(t\right)$ are, respectively, the random regression coefficients of additive and permanent environment effects, $e_{\mathrm{ijklm}}$ is the residual random effect. Regression curves were modeled using Legendre polynomials of order 2. Residual random effects were assumed to be normally distributed with a mean of 0.0. Homogeneity of residual variance was tested for SCS by computing the standard deviation (SD) of observed residuals (difference between observed and estimated values) for each DIM in the first three parities. The (co)variance components were estimated by Bayesian inference using the Gibbs sampler of the GIBBS3F90 program [10]. Gibbs sampling was used to obtain marginal posterior distributions for the various parameters using a single chain of 200,000 iterates. The first 50,000 iterates of each chain were regarded as a burn-in period to allow sampling from the proper marginal distributions. The length of the burn-in period was determined by visually inspecting plots of sample values across rounds. Daily heritability was defined as the ratio of genetic variance to the sum of genetic, permanent environmental, and residual variances at a given DIM.

## 3. Results

**Figure 1:**
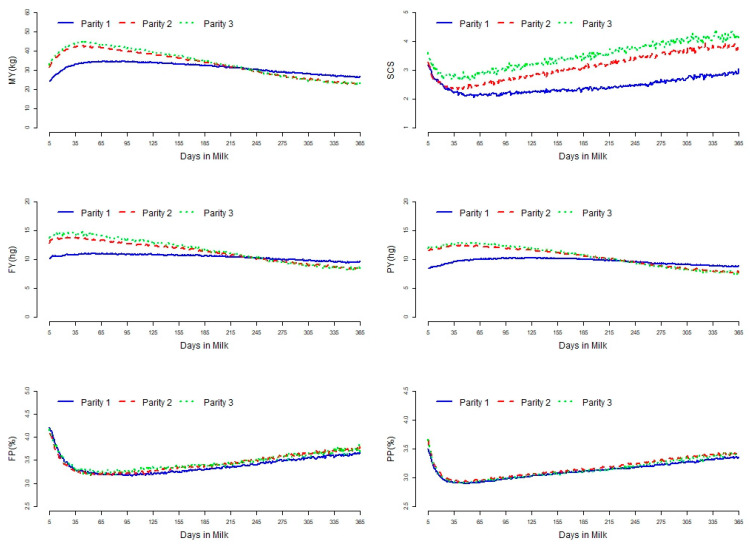
Lactation curve for daily milk yield (MY), SCS, fat yield (FY), protein yield (PY), fat percentage (FP), and protein percentage (PP) in the first three parities.

Descriptive statistics of the dataset used for parameter estimation for all traits included are shown in Table 1. The average level of SCS increased with increasing parity having means (SD) of 2.39 (1.97), 2.99 (2.06), and 3.37 (2.11) in the first, second, and third parities, respectively. The average daily milk yield was 31.6 kg with 3.36% of fat and 3.09% of protein in the first parity. The corresponding values for the second and third parities were 34.7 kg, 3.40%, and 3.13% and 35.8 kg, 3.42%, 3.10%. Figure 1 shows trajectories of SCS along with those for MY, FY, PY, FP, and PP by DIM for each parity. Lower SCS was found at the time of the milk yield peak, then increased for the remainder of the lactation. The lactation curves for MY showed that the first parity was more persistent but the second and third parities had higher peak yields to decline more thereafter compared to the first parity. Lactation curves for FY and PY had the same pattern as those for MY. However, in the latter part of the lactation, average first-parity yield traits (MY, FY, and PY) were higher compared to higher parities.

### 3.1. Variance Components

**Figure 2:**
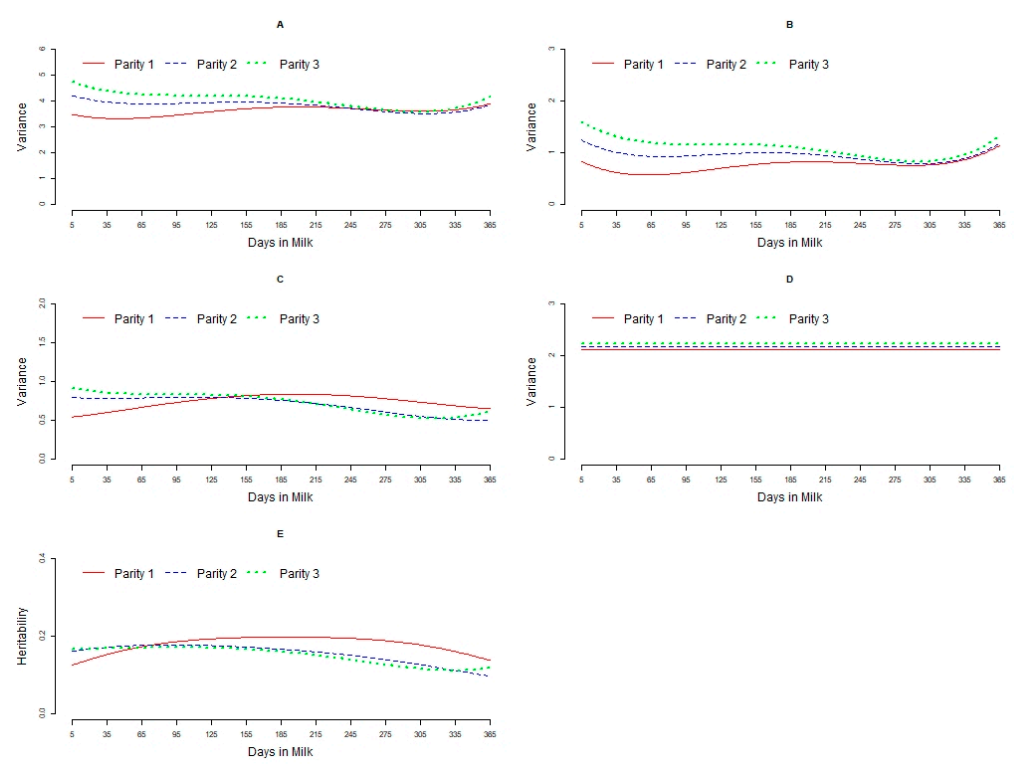
Phenotypic (**A**), permanent environmental (**B**), additive genetic (**C**), residual variance (**D**), and heritability (**E**) estimated for SCS over days in milk in the first three parities.

The phenotypic, permanent environmental, genetic, and residual variances by DIM for SCS in each parity are presented in Figure 2. Phenotypic, permanent environmental, and residual variance found for SCS increased with increasing parity. The average phenotypic variances by DIM for the first, second, and third parities were, respectively, 3.59, 3.80, and 4.02. The average permanent environmental variances found for SCS by DIM for the first, second, and third parities were, respectively, 0.75, 0.93, and 1.09. The corresponding values for the residual variance were, respectively, 2.10, 2.17, and 2.22. The genetic variance found for SCS in the first parity (0.74) was higher than those found for the second (0.70) and third parities (0.71). A high difference in phenotypic and permanent environmental variances was found between parities at the beginning of the lactation. This difference decreased with increasing DIM and disappeared at the end of the lactation. The observed genetic variance found in the first parity compared to those found in later parities was lower at early lactation, but higher in late lactation. The pattern of genetic variance by DIM found in the second parity was similar to that found for the third parity.

### 3.2. Heritabilities

The patterns of estimated heritabilities on a daily basis for SCS in the first three parities are represented in Figure 2. The results showed that h^2^ of SCS in the first parity compared to those for higher parities was lower in early lactation, but higher in mid and late lactation. The heritability pattern found in the second parity was the same as that found in the third parity. The mean (range) heritability estimates across lactation for SCS were 0.18 (0.11 to 0.20), 0.15 (0.11 to 0.17), and 0.15 (0.12 to 0.17), respectively, in the first, second, and third parities.

### 3.3. Relationships between Traits

**Figure 3:**
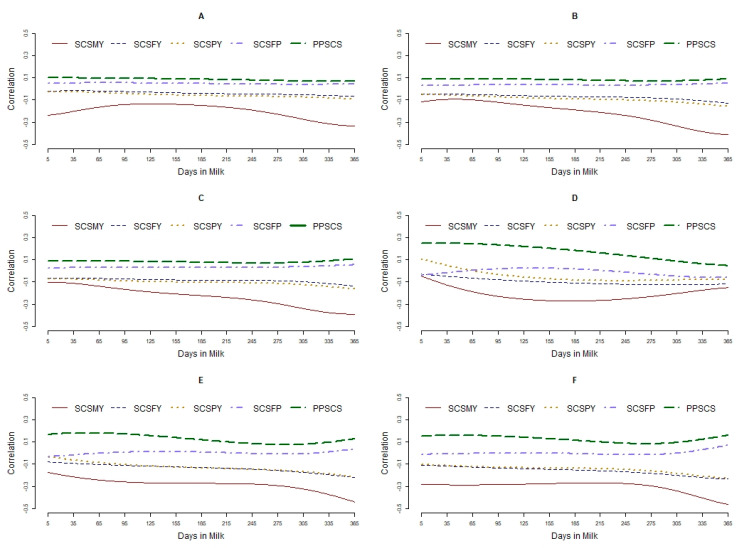
Phenotypic and genetic correlations estimated between SCS and productive traits over days in milk in the first three parities. Phenotypic correlation in the first parity (**A**), second parity (**B**), and third parity (**C**). Genetic correlation in the first parity (**D**), second parity (**E**), and third parity (**F**). SCSMY, SCSFY, SCSPY, SCSFP, and SCSPP, are abbreviations for somatic cell score and milk yield (SCSMY), fat yield (SCSFY), protein yield (SCSPY), fat percentage (SCSFP), and protein percentage (SCSPP), respectively.

The average phenotypic, genetic, and permanent environmental correlations between SCS and production traits are presented in Table 2, Table 3 and Table 4, respectively. The patterns of phenotypic and genetic correlations estimated on a daily basis between SCS and production traits included are presented in Figure 3. Weak negative phenotypic correlations ranging from −0.04 to −0.27 were found between SCS and yield traits (MY, FY, PY); however, weak positive phenotypic correlations ranging from 0.03 to 0.08 were found between SCS and FP and PP. The mean (range) genetic correlations between SCS and MY in the first three parities were, respectively, −0.21 (−0.27 to −0.04), −0.28 (−0.47 to −0.17), and −0.30 (−0.45 to −0.27). The corresponding values found between SCS and FY were −0.10 (−0.13 to −0.03), −0.14 (−0.23 to −0.08), and −0.16 (−0.22 to −0.11), and those found between SCS and PY were −0.05 (−0.09 to 0.11), −0.13 (−0.23 to −0.04), and −0.15 (−0.22 to −0.10). The mean (range) genetic correlations between SCS with PP in the first three parities were, respectively, 0.17 (0.05 to 0.25), 0.12 (0.07 to 0.17), and 0.13 (0.09 to 0.18). The corresponding values found between SCS and FP were −0.01 (−0.06 to 0.02), 0.00 (−0.03 to 0.05), and −0.00 (−0.4 to 0.06). For primiparous cows, the lowest genetic correlation between SCS and MY was found in the mid-lactation. The genetic correlation between SCS and MY in primiparous cows was lower at the mid-lactation compared to correlations found at the beginning and end of the lactation. The genetic correlation between SCS and MY in multiparous cows was high at the beginning of lactation, decreased with increasing DIM, and was the lowest at the end of lactation.

## 4. Discussion

Lower SCS was found at peak milk yield, then increased for the remainder of the lactation which is in close agreement with previous studies [8,11]. The average level of SCS increased with increasing parity which agrees with previous studies [11,12,13,14]. An increase in the SCS with increasing parity can be attributed to the fact that older cows have more opportunity for exposure to mastitis-causing pathogens [15,16,17]. The average heritabilities estimated for SCS in this study were 0.18, 0.15, and 0.15 for the first, second, and third parity, respectively, which are slightly higher than those reported in previous studies [8,18,19]. Miglior et al. [8] reported that the average daily heritabilities for SCS in Chinese Holstein were 0.09, 0.15, and 0.19, for the first, second, and third parity, respectively. Haile-Mariam et al. [12] reported that average daily heritability for SCS in Australian dairy cattle ranged from 0.09 to 0.11 in the first three parities. The results showed that average phenotypic correlations between SCS and yield traits ranged from −0.04 to −0.27. The genetic correlations found between milk yield and SCS ranged from −0.21 to −0.30, in the first three parities. It is well documented that the correlation between milk yield and SCS varies both within and across parities [11]. The negative relationships found between SCS and yield traits are consistent with the deleterious effect of poor mammary health on production [20]. Franzoi et al. [21] reported that an increase in SCC impairs lactation performance. An elevated SCS may indicate a bacterial infection of the udder which could have an adverse effect on milk production explaining the negative relationship between SCS and MY found in this study. In addition, higher MY may cause a decrease in SCS at the same level of infection through dilution effects, assuming that the SCS is proportional to the degree of infection. Miglior et al. [8] reported that the genetic correlations between milk yield and SCS were −0.12, −0.20, and −0.36, respectively, in the first, second, and third parities in Chinese Holstein cows. Koivula et al. [22] reported a positive genetic correlation between SCC and yield traits in the first parity, and near zero in the second parity in Ayrshire dairy cows in Finland.

## 5. Conclusions

Genetic parameters were estimated for SCS in Iranian Holstein dairy cows using a random regression test-day model. SCS had a low negative correlation with the production traits, but its magnitude increased in later parities. The moderate heritability for SCS and its low negative genetic correlations with yield traits indicates that genetic selection for decreasing SCS would have a relatively medium genetic progress with no necessarily antagonistic effects on lactation performance. The estimates found in this study can be considered as the first step to include SCS in the national genetic evaluations in Iranian Holsteins using a multiple-trait, multiple-lactation random regression model.

## Figures and Tables

**Table 1:** Descriptive statistics for milk yield and compositions for the first three parities in Holstein dairy cows in Iran.

	First Parity			Second Parity			Third Parity		
Traits	Mean	SD	CV (%)	Mean	SD	CV (%)	Mean	SD	CV (%)
Milk yield	31.6	7.6	24	34.7	10.4	30	35.8	11.3	32
Fat yield ^1^	10.49	3.34	32	11.63	4.34	37	12.09	4.72	39
Protein yield ^2^	9.70	2.38	24	10.73	3.10	29	10.96	3.35	31
Fat percentage	3.36	0.83	25	3.40	0.88	26	3.42	0.89	26
Protein percentage	3.09	0.39	13	3.13	0.41	13	3.10	0.41	13
SCS ^3^	2.39	1.97	82	2.99	2.06	68	3.37	2.11	63

^1^ Daily fat yield (hg), ^2^ daily protein yield (hg), ^3^ SCS = log_2_ (SCC/100,000) + 3.

**Table 2:** Mean (range) phenotypic correlations between SCS and production traits in the first three parities of Iranian Holstein cows.

	First Parity	Second Parity	Third Parity
Milk yield	−0.19 (−0.00 to −0.00)	−0.25 (−0.00 to −0.00)	−0.27 (−0.00 to −0.00)
Fat yield	−0.04 (−0.00 to −0.00)	−0.08 (−0.00 to −0.00)	−0.09 (−0.00 to −0.00)
Protein yield	−0.06 (−0.00 to −0.00)	−0.10 (−0.00 to −0.00)	−0.11 (−0.00 to −0.00)
Fat percentage	0.05 (−0.00 to −0.00)	0.04 (−0.00 to −0.00)	0.03 (−0.00 to −0.00)
Protein percentage	0.08 (−0.00 to −0.00)	0.08 (−0.00 to −0.00)	0.08 (−0.00 to −0.00)

**Table 3:** Mean (range) genetic correlations between SCS and production traits in the first three parities of Iranian Holstein cows.

	First Parity	Second Parity	Third Parity
Milk yield	−0.21 (−0.27 to −0.04)	−0.28 (−0.47 to −0.17)	−0.30 (−0.45 to −0.27)
Fat yield	−0.10 (−0.13 to −0.03)	−0.14 (−0.23 to −0.08)	−0.16 (−0.22 to −0.11)
Protein yield	−0.05 (−0.09 to 0.11)	−0.13 (−0.23 to −0.04)	−0.15 (−0.22 to −0.10)
Fat percentage	−0.01 (−0.06 to 0.02)	0.00 (−0.03 to 0.05)	0.00 (−0.0 to 0.09)
Protein percentage	0.17 (0.05 to 0.25)	0.12 (0.07 to 0.17)	0.13 (0.09 to 0.18)

**Table 4:** Mean (range) permanent environmental correlations between SCS and production traits in the first three parities of Iranian Holstein cows.

	First Parity	Second Parity	Third Parity
Milk yield	−0.16(−0.29 to −0.10)	−0.21(−0.42 to −0.10)	−0.24(−0.42 to −0.10)
Fat yield	−0.04(−0.11 to −0.00)	−0.10(−0.21 to −0.01)	−0.11(−0.21 to −0.03)
Protein yield	−0.06(−0.11 to −0.02)	−0.09(−0.21 to −0.02)	−0.10(−0.19 to −0.02)
Fat percentage	0.06(0.02 to 0.09)	0.02(0.00 to 0.04)	0.02(−0.01 to 0.05)
Protein percentage	0.05(0.03 to 0.08)	0.07(0.05 to 0.08)	0.03(0.05 to 0.12

## Data Availability

Relevant information supporting the results not presented here is provided in supplemental files (https://github.com/hadiatashi/Atashi-et-al-2021-SCS/SCS.rar) (26 May 2021).
